# High incidence of HPV infection in minors with oral squamous cell carcinoma

**DOI:** 10.1186/s13000-024-01470-9

**Published:** 2024-03-09

**Authors:** Ningxiang Wu, Yonghui Li, Xiaokun Ma, Zhen Huang, Zhuoxuan Chen, Weihua Chen, Ran Zhang

**Affiliations:** 1 Department of Oral Pathology, Peking University School and Hospital of Stomatology, 22 South Zhongguancun Avenue, Haidian District, Beijing, 100081 China; 2 Research Unit of Precision Pathologic Diagnosis in Tumors of the Oral and Maxillofacial Regions, Chinese Academy of Medical Sciences, Beijing, China; 3 The Affiliated Stomatological Hospital, Jiangxi Medical College, Nanchang University, Nanchang, 330006 China; 4 Department of Oral and Maxillofacial Surgery, Peking University School and Hospital of Stomatology, Beijing, China; 5 Department of Oral and Maxillofacial Surgery, The Affiliated Stomatological Hospital of Soochow University, Suzhou Stomatological Hospital, Suzhou, China; 6 College of Life Sciences, Fujian Normal University, Fuzhou, China; 7 Jiangxi Province Key Laboratory of Oral Biomedicine, Nanchang, China; 8 Jiangxi Province Clinical Research Center for Oral Diseases, Nanchang, China

**Keywords:** Oral squamous cell carcinoma, Minors, HPV infection, Fluorescence in-situ hybridization

## Abstract

**Background:**

Oral squamous cell carcinoma in minors is considered to be a distinct entity from OSCC in older patients, with an uncertain etiology. Human papillomavirus (HPV) infection may trigger the initiation and promote the progression of OSCC, but these roles have not been firmly established.We aimed to explore the correlation between HPV infection and the development of oral squamous cell carcinoma in minors and know the characteristics of OSCC in young patients more thoroughly.

**Method:**

From January 2013 to December 2022,6 cases of OSCC aged < 15 years were selected from the Department of Oral Pathology, Peking University School of Stomatology, Beijing, China. All cases underwent testing for high-risk HPV mRNA infection using the RNA scope technique, and immunohistochemical staining was performed to investigate the expression of p16, pan-cytokeratin (CK), CK5/6, CK7, CK8/18, epidermal growth factor receptor (EGFR), p53, and Ki-67. Furthermore, we reviewed the literature on OSCC in patients aged < 21 years.

**Conclusions:**

Minors OSCC is associated with HPV infection, and that p16 can serve as an immunohistochemical marker of HPV positivity.

## Introduction

Oral squamous cell carcinoma (OSCC) is a malignant tumor of epithelial origin typically found in the oral cavity that displays varying levels of squamous differentiation, as evidenced by the formation of keratinized beads and intercellular bridges. This type of cancer is most common in middle-aged and elderly individuals, less prevalent in those aged ≤ 40 years, and exceedingly rare in those aged < 15 years [1–3]. OSCC is generally linked to tobacco and alcohol consumption, and these risk factors are not commonly present in minors. Thus, OSCC in minors is considered to be a distinct entity from OSCC in older patients, with an uncertain etiology. Moreover, human papillomavirus (HPV) infection may trigger the initiation and promote the progression of OSCC, but these roles have not been firmly established.

The treatment of OSCC typically involves a comprehensive approach, with surgical removal followed by chemotherapy and radiation therapy [4]. Some studies have revealed that the prognosis of OSCC is worse for minors than for older patients, whereas others suggest that survival rates in these groups are similar. Given the rarity of adolescent OSCC, most reports describe small numbers of cases. To explore the characteristics of OSCC in young patients more thoroughly, we examined the clinical and pathological features, treatment, and prognosis of six cases diagnosed at our hospital in patients aged ≤ 15 years. All cases underwent testing for high-risk HPV mRNA infection using the RNA scope technique, and immunohistochemical staining was performed to investigate the expression of p16, pan-cytokeratin (CK), CK5/6, CK7, CK8/18, epidermal growth factor receptor (EGFR), p53, and Ki-67. Furthermore, we reviewed the literature on OSCC in patients aged < 21 years. Three cases displayed concurrent positive expression of HPV16/18 and p16. Our analysis of the clinicopathological and HPV mRNA data suggests that adolescent OSCC is associated with HPV infection, and that p16 can serve as an immunohistochemical marker of HPV positivity.

## Materials and methods

### Patients

We reviewed OSCC cases in patients aged < 15 years that were diagnosed between January 2013 and December 2022 at the Department of Oral Pathology, Peking University School of Stomatology, Beijing, China. A senior pathologist validated the clinical and pathological data for these cases following surgical resection. Data on patient age and sex, tumor location, clinical manifestations, treatment, and prognosis were extracted from medical records. Of the six cases examined, one was a recurrence and five were new lesions.

## Methods

### Immunohistochemistry

 Specimens were fixed with 10% neutral buffered formalin before routine dehydration and paraffin embedding. Section (4 μm thick) were dewaxed with xylene and rehydrated with a series of ethanol concentrations, followed by washing with distilled water and phosphate-buffered saline (PBS). The sections were then microwaved with citrate repair solution for 3.5 min, cooled to room temperature, and washed three times with PBS. The EnVision two-step method was used for the detection of p16, pan-CK, CK5/6, CK7, CK8/18, EGFR, p53, and Ki-67 protein antibodies, with incubation at 37 °C for 30 min. The antibodies and staining parameters used were: p16 (ZM-0205, clone name 1C1; ZSGB-Bio, China), pan-CK (ZM-0069, clone name AE1/AE3; ZSGB-Bio), CK5/6 (ZM-0313, clone name OTI1C7; ZSGB-Bio), CK7 (ZM-0071, clone name UMAB161; ZSGB-Bio), CK8/18 (ZM-0315, clone names B22.1 and B23.1; ZSGB-Bio), p53 (ZM- 0408, clone name DO-7; ZSGB-Bio), EGFR (ZM-0088, clone name UMAB233; ZSGB-Bio), and Ki-67 (ZM-0167, clone name MIB1; ZSGB-Bio).

### Interpretation of immunohistochemical results

The staining sites of pan-CK, CK5/6, CK7, CK8/18 and EGFR were in cytoplasm [5]. If more than 80% of tumor nuclei show strong positive expression or no expression at all, it is indicative of a p53 mutant; conversely, if the tumor cells show positive expression in scattered nuclei or with varying levels of intensity, it is a p53 wild type [6]. Any intensity of nuclear staining indicates a Ki67 positive cell [7]. In the interpretation of the p16 immunohistochemistry results, tumors exhibiting strong, diffuse staining of the nuclei and cytoplasm of > 70% of cells were classified as positive, and all other tumors were classified as negative [8].

### Fluorescence in-situ hybridization and interpretation

Fluorescence in situ hybridization (FISH) was performed on 4-µm-thick sections derived from formalin-fixed paraffin-embedded tissues for HPV detection, according to the manufacturer’s instructions. The RNAscope HPV HR18 multi-subtype probe, designed to detect 18 high-risk HPV subtypes (HPV16, -18, -26, -31, -33, -35, -39, -45, -51, -52, -53, -56, -58, -59, -66, -68, -73, and − 82), was used. Cells were examined under a fluorescence microscope; green dot-like signals indicated positivity and their absence indicated negativity.

Two oral pathology experts independently assessed all FISH and immunohistochemical results.

## Results

### Clinical and radiological findings

The study sample comprised four females and two males aged 3–14 (mean, 9.8; median, 11) years. Five cases presented as primary lesions and one presented as a recurrence. Two lesions were located in the upper and lower gingiva, respectively, and the remaining four lesions were located in the mandible, maxilla, left submandibular region, and left tongue, respectively. One patient had a history of aplastic anemia, bone marrow transplantation, and previous HPV infection. The other patients had no systemic disease or family history of malignancy.

**Fig. 1:**
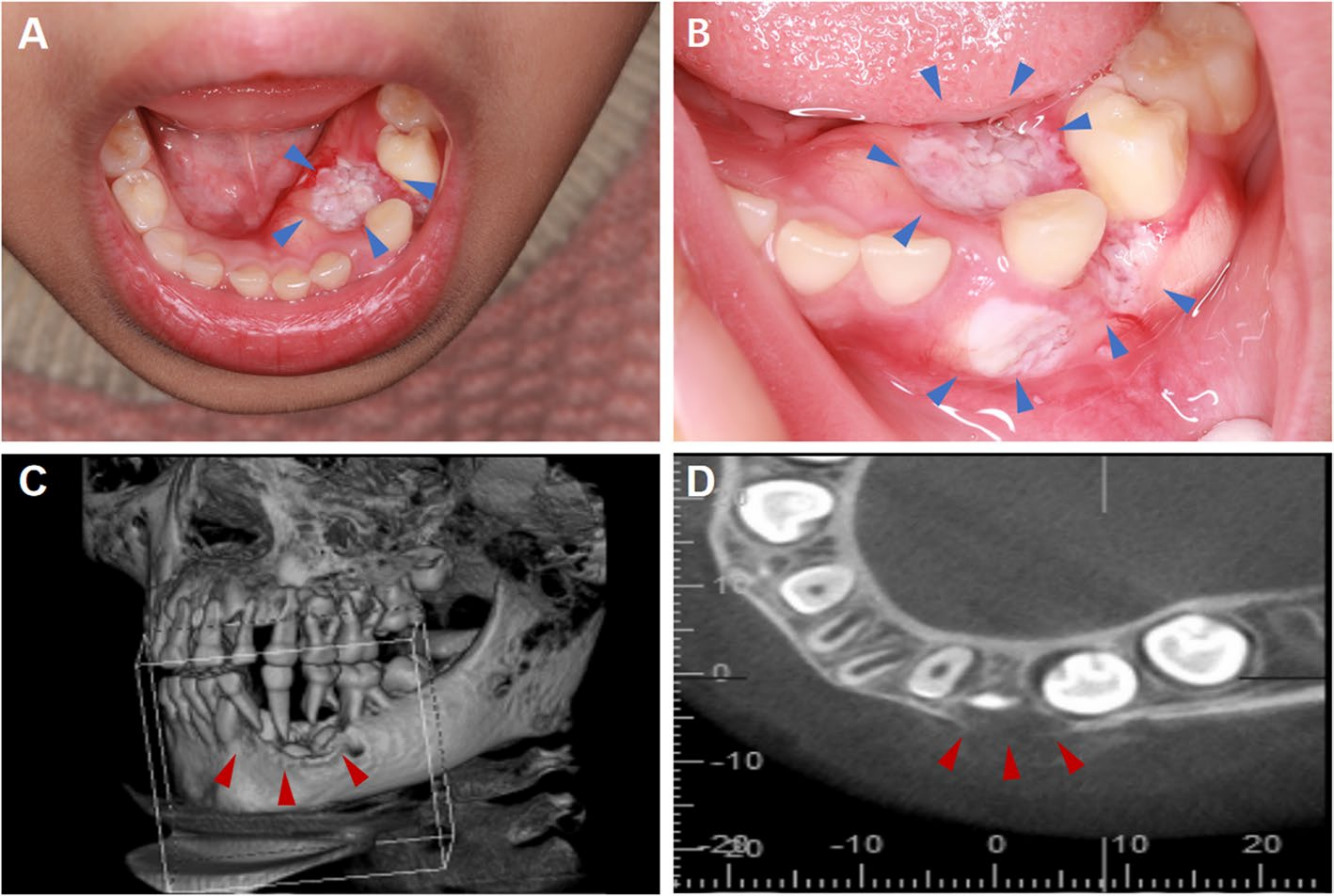
Clinical characteristics and radiological features of OSCC. **A** and **B** Clinical photograph of left mandibular gingival lesion at presentation shows cauliflower-shaped lesions on the buccal and lingual side of deciduous mandibular left canine tooth (blue arrows indicate). **C** and **D** Cone Beam Computed Tomography (CBCT) showed that resorption and destruction of the bone around the roots of teeth 72 to 75 can be seen, with unclear borders and bone discontinuity at the perimeters (red arrows indicate). The buccal lingual cortex is discontinuous and visible periosteal reactions are present. Additionally, the roots of 73 and 74 teeth are floating and no external absorption is visible (red arrows indicate)

Four cases presented primarily as lumps and two cases presented as ulcers (Fig. 1A, B). Five patients underwent spiral computed tomography examinations; one patient did not undergo imaging examination at our hospital. The scans revealed bone destruction in two cases (Fig. 1C, D), multiple abnormal lymph nodes in the neck in two cases, a malignant tumor in the left tongue and floor of the mouth in one case, and decreased bone density with visible marginal incisions in one case. Five patients had received initial medical care at local facilities, including initial surgical procedures in four cases. Biopsy results indicated that the lesions were squamous cell carcinoma in two of those cases and squamous epithelial tumor-like hyperplasia in the other two cases.

Postoperatively, two patients reported pain and lack of wound healing. One patient developed nonlocal recurrence 2 months after surgery. All patients treated at our hospital underwent surgical procedures (i.e., osteotomy, extended resection). One patient developed recurrence 1 year after the initial surgery, but not after a second surgery. One patient was lost to follow-up. The remaining patients showed no evidence of recurrence. The follow-up period ranged from 3 months to 5 years. Detailed clinical information is presented in Tables 1 and 2.

**Table 1:** Clinical characteristics of OSCC in adolescents

Case no.	Age, y/sex	Location	Clinical manifestation	Size/cm	Management	LN metastasis	Radiotherapy/chemotherapy	Follow-up	TNM stage
1	6/F	Lower gingiva	Mass	1.5 × 1.5 × 1.5	ER + Segmental mandibulectomy + CLND	No	Yes	NR, 3 mo	T2N0M0
2	14/M	Upper gingiva	Ulceration	5 × 3 × 3	ER + Hemi-maxillectomy + CLND	Yes	Yes	1st R, 1 y; 2nd NR	T3N2bM0
3	14/M	Right mandible	Mass	3 × 2.5 × 2	ER + Segmental mandibulectomy + CLND	No	No	NR, 2 y	T3N0M0
4	3/F	Submandibular triangle	Mass	7 × 8 × 8	Enucleation + CLND	No	N/A	/	T3N0M0
5	14/F	Tongue	Ulceration	3.5 × 2.5 × 1.5	ER + CLND	No	N/A	NR, 18 mo	T2N0M0
6	8/F	Left maxilla	Mass	N/A	ER + Hemi-maxillectomy + CLND	No	N/A	NR, 5 y	N/A

*F* female, *M* male, *y* year, *mo* month, *ER* extended resection, *CLND* cervical lymph node dissection, *LN* lymph node metastasis, *R* recurrence, *NR* no recurrence, *NA* not available, */* loss to follow-up

**Table 2:** Pathological and molecular findings of oral squamous cell carcinoma in adolescents

Case no.	Grade	IHC P16	FISH
1	I	+	+
2	II~III	+	+
3	I	-	-
4	II~III	-	-
5	I	-	-
6	I	+	+

*IHC* immunohistochemistry, *FISH* fluorescence in-situ hybridization

### Histopathological findings

**Fig. 2:**
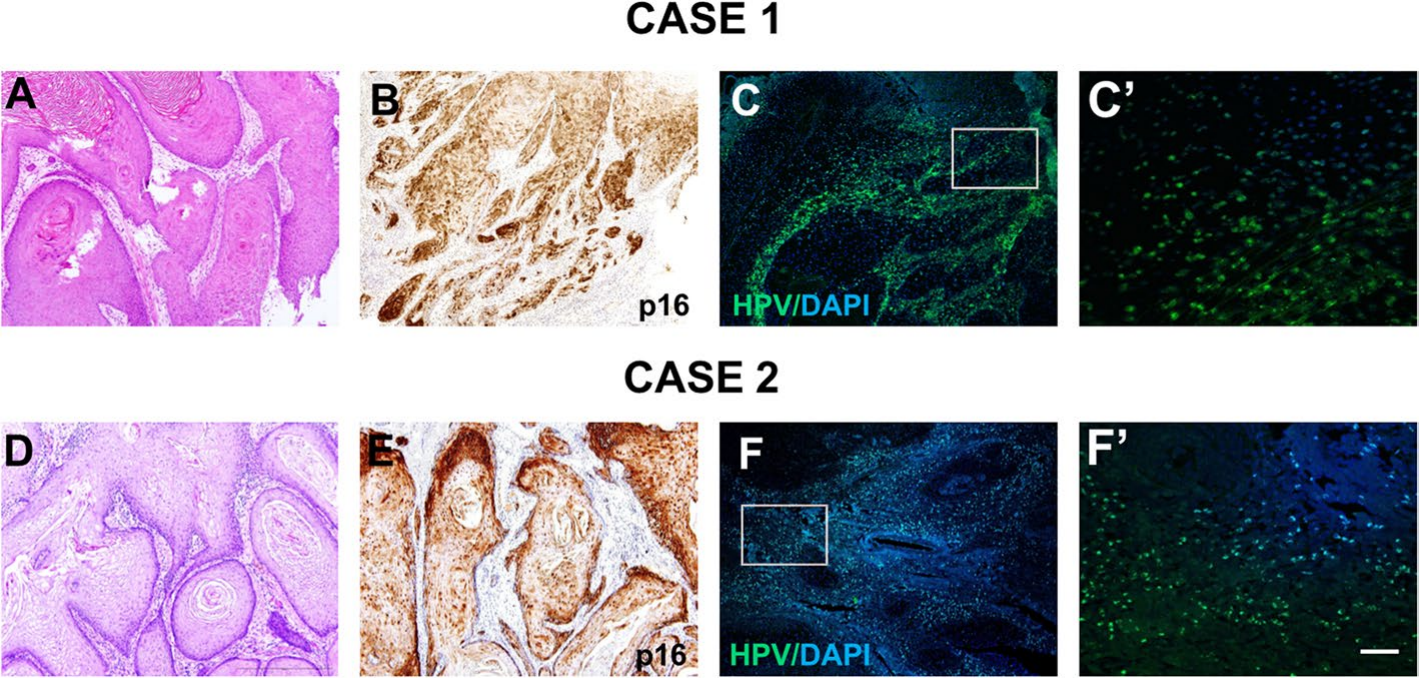
Representative images of histological characteristics, immunohistochemical staining and FISH. **A** and **D** Excised tissue stained with hematoxylin and eosin showing the histologic appearance of well-differentiated and middle differentiated squamous cell carcinoma. **B** and **E** Positive immunostaining for p16 in OSCC. **C** and **F** Examples of HPV mRNA detection with FISH showing yielded positive results in OSCC. (C’ and F’ are higher-magnification views of boxed areas) Scar Bar: 500 μm (**A**, **B**, **D** and **E**) 0.100 μm (**C**, **F**), 25 μm (C’, F’)

The cancer tissues consisted of differently sized nests with keratinized pearls congregating at the nest centers. The squamous epithelium of the lesions extended irregularly, breaching the basement membrane and infiltrating the subcutaneous connective tissue. Tumor cells varied in size and displayed eosinophilic cytoplasm with a range of squamous differentiation. Abnormal mitotic figures were frequently observed. Squamous cell carcinoma can be categorized as grade I (characterized by keratinization, few mitotic figures, and minimal cell pleomorphism), grade II (infrequent keratinization with the presence of abnormal mitotic figures), and grade III (scarcity of keratinization and abundance of abnormal mitotic figures). Four of our cases were grade I (Fig. 2A) and two were grade II–III (Fig. 2D). One case presented with lymph node metastasis and another invaded the gland, nerve, and muscle tissue. All cases were negative for extranodal extension (ENE) and positive for pan-CK, CK5/6, and EGFR. Depths of invasion were 8.9–20 mm. Three cases were positive for p16 (Fig. 2B, E), whereas few basal layer cells and cancer nest tissues expressed p53. Ki-67 proliferation indices were 10–30%, and CK8/18 (1/6, 16.7%) was detected. All patients were negative for CK7. HPV mRNA detection with FISH, considered to be definitive for the detection of HPV infection in patients with OSCC, was conducted in all six cases and yielded positive results in three cases, consistent with the p16 expression pattern (Fig. 2C’).

### Literature review

Table 3 summarizes the clinical characteristics of 181 patients aged < 21 years with OSCC, extracted from the related literature. Two cases were documented in newborns. Of the patients whose ages were reported, 99 were aged ≥ 15 years and 68 were aged < 15 years. The majority (55.3%) of patients were male. The tongue was the most common site of onset, observed in 55.8% of cases, followed by the gingiva (10.5% of cases). Our sample comprised four females and two males with lesions in the tongue, gingiva, maxilla, mandible, and submandibular area. One patient was believed to have a metastasis of squamous cell carcinoma of the left mandible.

**Table 3:** Summary of reported oral squamous cell carcinoma cases in patients aged < 21 years

Author	Year	No. of cases	Age (y)	Sex	Site
Frank et al. [9]	1936	1	Newborn	M	Tongue
Saleeby et al. [10]	1940	1	15	F	Tongue
Frazell and Lucas [11]	1962	1	19	F	Lateral tongue
Venables and Craft [12]	1967	1	17	F	R border of tongue
Goyanes [13]	1971	6	19	F	R side of tongue
			18	M	L side of tongue
			18	F	R side of tongue
			16	M	L side of tongue
			19	F	R side of tongue
			16	F	R lateral border of tongue
Pichler et al. [14]	1972	1	19	M	R lateral border of tongue
Turner and Snitzer [15]	1974	1	12	M	R dorsal, ventral tongue
Byers [16]	1975	4	17		Tongue
			19	NS	
			19		
			19		
Krolls and Hoffman [17]	1976	19	14	M	Tongue
			14	F	Tongue
			14	M	Low lip
			15–19 (16)	NS	NS
Patel et al. [18]	1976	5	16	M	R side of anterior tongue
			17	M	R side of tongue
			16	M	Tongue
			11	M	R side of neck and tongue
			11	M	Tongue
Harper et al. [19]	1981	1	18	M	Tongue
Yagi et al. [20]	1981	1	10	F	Tip of tongue
Kennedy [21]	1982	1	20	F	L lateral margin of tongue
Nuutinen and Sainio [22]	1982	1	9	F	L lateral margin of tongue
Newman et al. [23]	1983	3	14		
			16	NS	Tongue
			18		
McGregor [24]	1983	2	< 20	F	Tongue
			18	F	Tongue
Son and Kapp [25]	1985	4	10	M	R maxillary alveolus
			17	F	R buccal
			18	M	R anterior tongue
			19	M	L anterior tongue
Kaplan et al. [26]	1985	3	8	F	Tongue
			14	M	Tongue
			7	F	Tongue
Sacks et al. [27]	1985	1	13	M	Maxillary alveolar mucosa
Usenius et al. [28]	1987	3	14	M	L lateral tongue
			7	F	Tip of tongue
Earle et al. [29]	1988	1	10	F	Tip of tongue
			7	M	R anterior maxillary gingiva
Kärjä et al. [30]	1988	3	14	M	Tongue
			7	F	Tongue
			10	F	Tongue
Amichetti et al. [31]	1989	1	14	F	Tongue
Lund et al. [32]	1990	1	20	NS	Tongue
Murayama et al. [33]	1990	1	11	M	L side of tongue
Socie et al. [34]	1991	1	6	M	Tongue
Morehead et al. [35]	1993	1	12	M	Tongue
Tsukuda et al. [36]	1993	4	10–19	F (2) M (2)	NS
Sarkaria et al. [37]	1994	1	17	M	Tongue
Somers et al. [38]	1995	1	16	M	Lateral edge of tongue
Atula et al. [39]	1996	2	19	M	Tongue
			19	F	Tongue
de Carvalho et al. [40]	1998	3	6	F	Gingiva
			14	M	Gingiva
			15	F	Soft palate
Jin et al. [41]	1999	2	6.5	F	Lower lip
			19	M	Lower lip
Myers et al. [42]	2000	1	16	NS	Tongue
Torossian et al. [43]	2000	1	13	M	R side of tongue
Oliver et al. [44]	2000	1	20	F	R lateral border of tongue
Pitman et al. [45]	2000	1	16	NS	Tongue
Bill et al. [46]	2001	1	14	M	L mandibular premolar gingiva
Soni et al. [47]	2001	1	8	F	Base of tongue
Wang et al. [48]	2001	1	20	M	Tongue
Abdelsayed et al. [49]	2002	1	14	M	L lateral border of tongue
Annertz et al. [50]	2002	1	20	NS	Tongue
Hyam et al. [51]	2003	1	19	NS	Tongue
Veness et al. [4]	2003	1	19	M	Anterior tongue
Lamaroon et al. [52]	2004	1	17	NS	NS
Sturgis et al. [53]	2005	3	18	M	Tongue
			16	F	Tongue
			20	F	Tongue
Sasaki et al. [54]	2005	1	19	F	NS
Salum et al. [55]	2006	1	12	M	L side of tongue
O’Regan et al. [56]	2006	2	16–20	M F	NS
Ajayi et al. [57]	2007	1	NS	M	Gingiva
Chow et al. [58]	2007	4	16	F	Tongue
			15	M	Tongue
			14	M	Tongue
			11	F	Cheek/Palate
Reinhard et al. [59]	2007	1	13	F	L side of tongue
Binahmed et al. [60]	2007	1	10	F	Gingiva
Stolk-Liefferink et al. [61]	2007	1	11	M	Gingiva
Effiom et al. [62]	2008	19	1st decade-3 2nd decade-16	M (15) F (4)	NS
Massaro et al. [63]	2008	3	17	M	Tongue
			17	F	Tongue
			10	M	Tongue
Randhawa et al. [64]	2008	1	19	F	R posterolateral border of tongue
Mehanna et al. [65]	2008	1	10	M	Gingiva
Alsharif et al. [66]	2009	1	16	F	Anterior mandibular gingiva
Woo et al. [3]	2009	4	11	F	R mandible gingiva
			11	F	R anterior maxilla gingiva
			17	M	L post maxilla gingiva
			18	F	Anterior mandible gingiva
Sidell et al. [67]	2009	1	6	M	Gingiva
Bachar et al. [68]	2010	1	15	NS	Tongue
Hutton et al. [69]	2010	1	7	M	Gingiva
Fadoo et al. [70]	2010	1	11	F	Tongue
Nagy et al. [71]	2010	1	15	F	Tongue
Morris et al. [72]	2010	10	15–20	F (8) M (2)	Tongue
Moubayed et al. [73]	2011	1	Newborn	F	Lower lip
Ribeiro et al. [74]	2011	1	7	M	Gingiva
Harirchi et al. [75]	2012	1	15	F	Base of tongue
Smith et al. [76]	2014	1	6	M	L mandible
Bhanuprasad et al. [77]	2015	10	10	M	Mandible
			12	F	Soft palate
			15	M	Gingiva
			15	M	Tongue
			17	M	Soft palate
			17	M	Hard palate
			19	M	FOM
			20	M	Tongue
			20	M	Tongue
			20	M	Buccal mucosa
Magalhaes et al. [1]	2016	1	8	M	L maxillary ridge
Chaudhary et al. [2]	2016	1	12	M	L lateral tongue
Kajal et al. [78]	2019	1	12	F	Tongue
Ambele et al. [79]	2019	1	19	F	L lateral border of tongue
David L et al. [80]	2020	3	16	F	R ventral lateral tongue
			18	F	L lateral border of tongue
			20	M	R lateral tongue
de Mendonça et al. [81]	2021	1	17	F	Tongue
Kim CS et al. [82]	2021	1	3	F	Tongue
Pereira et al. [83]	2021	1	20	M	Lower lip
Medeus et al. [84]	2022	1	14	M	Tongue
Şahin et al. [85]	2022	1	7	M	Upper lip
Yabe et al. [86]	2022	1	19	F	R tongue
Kikuta et al. [87]	2023	1	19	F	Gingiva

*F* female, *M* male, *y* year, *R* right, *L* left, *FOM* floor of mouth, *NS* not specific

## Discussion

OSCC is the predominant malignant tumor in the oral cavity, constituting almost 90% of all oral malignancies [17]. Its incidence is highest among individuals aged 60–80 years, with a slightly higher prevalence in males [87]. The occurrence rate is significantly lower in individuals aged ≤ 40 years and even lower in those aged ≤ 15 years. In an emerging trend, however, OSCC is being diagnosed at younger ages. In 1936, Frank et al. [9] reported a case of tongue squamous cell carcinoma in an infant, the youngest patient with this malignancy known to date, who underwent radiation therapy. In 2011, Moubayed et al. [73] documented a case of squamous cell carcinoma in a newborn’s lips. Okuyama et al. [88] analyzed the clinical and pathological characteristics of OSCC in 107 patients aged 15–39 years and 420 patients aged ≥ 70 years; they found that the tongue was most commonly affected in both groups and the floor of the mouth was also prone to lesions, with no significant sex difference.

Smoking and alcohol consumption are recognized risk factors for OSCC development in adults [52, 73, 89], whereas these risk factors are infrequently present in minors. No patient in our sample had a history of smoking, drinking, or betel nut chewing. Several studies have suggested that squamous cell carcinoma development is more likely in children with conditions such as Fanconi anemia, xeroderma pigmentosum, and keratitis-ichthyosis-deafness syndrome [60, 80]. Individuals with xeroderma pigmentosum face a 3000-fold increased risk of developing tongue cancer compared with the general population [78]. Harper et al. [19] reported a case of severe progressive xeroderma pigmentosum in an 18-year-old male who died of verrucous carcinoma of the tongue. Yagi [20] also documented a case of xeroderma pigmentosum that led to squamous cell carcinoma of the tongue in a man whose brother had the same skin condition. Research indicates that bone marrow transplantation increases the risk of solid tumor development [55]. Socie et al. [34] reported that 4 of 147 patients with anemia (107 with aplastic and 40 with Fanconi anemia) who underwent bone marrow transplantation between 1980 and 1989 had developed malignant solid tumors after 64 months of follow-up. Masserot et al. [63] reported that 13 patients with Fanconi anemia developed head and neck squamous cell carcinoma after stem cell transplantation. Notably, one patient in our study with aplastic anemia who underwent immunosuppressive therapy and bone marrow transplantation developed a tongue ulcer, later diagnosed as squamous cell carcinoma, 3 years thereafter.

In recent years, HPV has been associated with squamous cell carcinoma of the head and neck, especially oropharyngeal squamous cell carcinoma. High-risk HPV cancers are driven by major HPV oncogenes, E6 and E7, which promote uncontrolled cell growth and genomic instability by down-regulating tumour suppressor genes, p53 and retinoblastoma (Rb), respectively [90]. The HPV status of oropharyngeal squamous cell carcinoma lesions is considered to be an independent prognostic factor, with positivity associated with a higher 5-year survival rate and better prognosis [91–93]. HPV has also been implicated in other types of head and neck cancer, including laryngeal squamous cell carcinoma and oral squamous cell carcinoma. Research indicated that the incidence of HPV positive OSCC had increased significantly from 1973 to 2004, particularly among white and at younger ages, the prevalence is higher among men than among women [94]. In a study involving 47 OSCC patients under the age of 40, it was discovered that 15 of them were HPV positive. These patients exhibited a 5-year survival rate of 52% and a 10-year survival rate of 27.7%, which was greater than that of HPV negative patients [95]. In this study, we conducted immunohistochemical detection of p16 protein and FISH detection of HPV mRNA in all cases. Three of the six patients tested positive for both p16 and HPV mRNA, and one had a history of HPV infection, and two of them received postoperative radiotherapy and chemotherapy. To date, recurrence has not developed in these three patients. Some researchers believe that the presence of HPV in OSCC had been related to improved prognosis, especially when chemo or radiotherapy is used,probably because of the absence of field cancerization or enhanced chemoradiation sensitivity [95]. Nonetheless, the association between HPV infection and the prognosis of OSCC in minors remains unclear.

The diagnosis of OSCC in minors presents a significant challenge for clinicians and pathologists, primarily because the signs and symptoms of OSCC in minors often mimic those of other conditions. In addition, lesions may be detected not in the precancerous stage, but only after significant progression. Clinically, gingival inflammation in adolescent patients is frequently mistaken for an inflammatory disorder, and lesions on the tongue are often misinterpreted as ulcers or injuries. Moreover, persistent lesions or ulcers in the mouth are frequently overlooked, leading to misdiagnosis or missed diagnoses. Morris et al. [72] reported a 6-month delay of biopsy by dentists and oral surgeons in 1 of 10 tongue squamous cell carcinoma cases in individuals aged 15–20 years, resulting in the patient’s presentation with a T3 tumor. Such delays can result in severe complications, such as carcinoma, and delayed treatment. Thus, the early biopsy of suspect lesions is crucial, especially in patients with histories of immunosuppression, previous tumors, radiotherapy, and/or genetic predisposition to malignancies. In cases of uncertainty, consultation with experienced pathologists and, when necessary, the prompt performance of more extensive repeat biopsy are recommended to avoid unnecessary diagnostic delays [58]. Two patients in our sample presented to the hospital with postoperative wound healing failure and two presented with unhealed ulcers. Postoperative pathological examination led to the diagnosis of squamous cell carcinoma in all of these cases. Hence, the early biopsy of such lesions is vital for timely diagnosis and treatment [60].

The diagnosis of highly and moderately differentiated squamous cell carcinomas in minors can be challenging, and these lesions can be mistaken for pseudoepitheliomatous hyperplasia. This non-cancerous epithelial cell growth, which can occur due to inflammation or the presence of a tumor, is characterized by irregular epithelial bands extending into the underlying connective tissue. It may also present keratinized beads that appear to infiltrate the region. On microscopic examination, it may resemble highly differentiated squamous cell carcinoma due to the separation of the proliferative epithelium from the surface epithelium. However, cell heterogeneity is less evident and the nuclear-to-cytoplasmic ratio is not as high [46, 66]. In addition, squamous cell carcinoma of the jaw must be differentiated from keratinizing ameloblastoma, which can also exhibit significant keratinization on microscopic examination but lacks cell heterogeneity and mitosis. p53 and Ki-67 immunohistochemical staining can aid the diagnosis. Whereas p53 expression is reduced in keratinizing ameloblastoma, p53 immunoreactivity is high in OSCC. Furthermore, the Ki-67 proliferation index is higher for OSCC than for keratinizing ameloblastoma. The majority of ameloblastomas have been demonstrated to have BRAF V600E mutations [96].

OSCC is characterized by robust infiltration and the propensity to metastasize, and is often diagnosed at an advanced stage in middle-aged and elderly patients. The primary treatment for adult OSCC comprises a comprehensive approach including extensive tumor resection, selective neck lymph node dissection based on the presence of lymph node metastasis during surgery, and postoperative chemotherapy and radiotherapy [43, 47, 89, 97]. The use of radiotherapy to treat minors, who are in a critical stage of development, could negatively affect the soft and hard tissues in the maxillofacial region, potentially leading to facial asymmetry and even secondary malignant tumors. Consequently, comprehensive surgical resection is the preferred treatment for adolescent OSCC. Certain research suggests that postoperative radiotherapy significantly reduces local recurrence rates, prompting some experts to recommend its consideration along with proactive surgical treatment for younger patients [47]. All patients in our sample who underwent radical neck dissection to remove the primary tumor and local lymph nodes were diagnosed with OSCC. The prognostic value of factors such as regional lymph node metastasis, infiltration depth, and neural invasion has been evaluated [98]. Among our cases, one patient had lymph node metastasis with no ENE. This patient received postoperative adjuvant chemotherapy and radiotherapy, but developed recurrence 1year postoperatively; no recurrence has been noted since the second surgery. Throughout the follow-up period, patients underwent regular physical examinations and imaging studies to monitor recurrence. No evidence of recurrence was observed.

This study is constrained by the limited number of cases analyzed. For a better understanding of OSCC occurrence in minors, the collection of more samples and in-depth analysis of associated factors in future research are crucial.

In conclusion, these findings have found that there is a high prevalence of HPV infection among minors suffering from oral squamous cell carcinoma. Early biopsy, p16 immunohistochemical staining, and HPV mRNA detection are vital for the effective treatment and prognosis determination of OSCC.

## Data Availability

No datasets were generated or analysed during the current study.

## Abbreviations

OSCC: Oral squamous cell carcinoma
HPV: Human papillomavirus
ENE: Extranodal extension

## References

[1] Magalhaes MA, Somers GR, Sikorski P (2016). Unusual presentation of squamous cell carcinoma of the maxilla in an 8-year-old child[J]. Oral Surg Oral Med Oral Pathol Oral Radiol.

[2] Neena C, Deepak KG, Rajeev KV, et al. Squamous cell carcinoma of tongue in a 12-year-old child. Acta Otolaryngol Case Rep. 2016;1(1):87–9.

[3] Woo VL, Kelsch RD, Su L (2009). Gingival squamous cell carcinoma in adolescence. Oral Surg Oral Med Oral Pathol Oral Radiol Endod.

[4] Veness MJ, Morgan GJ, Sathiyaseelan Y (2003). Anterior tongue cancer: age is not a predictor of outcome and should not alter treatment. ANZ J Surg.

[5] Wang JH, Zhu H, Shang YF (2022). [Nasopharyngeal carcinoma with non-squamous immunophenotype: a clinicopathological analysis of 23 cases]. Zhonghua Bing Li Xue Za Zhi.

[6] Gencel-Augusto J, Lozano G (2020). p53 tetramerization: at the center of the dominant-negative effect of mutant p53. Genes Dev.

[7] Carr RA, Mesiano D, Heffron C (2023). Aberrant p16, p53 and Ki-67 immunohistochemistry staining patterns can distinguish solitary keratoacanthoma from cutaneous squamous cell carcinoma. Pathology.

[8] Prigge E, Arbyn M, von Knebel Doeberitz M (2017). Diagnostic accuracy of p16INK4a immunohistochemistry in oropharyngeal squamous cell carcinomas: a systematic review and meta-analysis. Int J Cancer.

[9] Frank LW, Enfield CD, Miller AJ (1936). Carcinoma of the tongue in a newborn child: report of a case. Am J Cancer.

[10] George WS. Cancer of the tongue in young subjects: with report of a case. Am J Cancer. 1940;38(2):225.

[11] Frazell EL, Lucas JC (1962). Cancer of the tongue. Report of the management of 1,554 patients. Cancer.

[12] Venables CW, Craft IL (1967). Carcinoma of the tongue in early adult life. Br J Cancer.

[13] Goyanes AD, Frazell EL (1971). Cancer of the tongue in young persons. J Surg Oncol.

[14] Pichler AG, Williams JR, Moore JA (1972). Carcinoma of the tongue in childhood and adolescence. Report of a case and review of the literature. Arch Otolaryngol (Chicago, Ill: 1960).

[15] Turner H, Snitzer J (1974). Carcinoma of the tongue in a child. Report of a case and review of the literature. Oral Surg Oral Med Oral Pathol.

[16] Byers RM (1975). Squamous cell carcinoma of the oral tongue in patients less than thirty years of age. Am J Surg.

[17] Krolls SO, Hoffman S (1976). Squamous cell carcinoma of the oral soft tissues: a statistical analysis of 14,253 cases by age, sex, and race of patients. J Am Dent Assoc.

[18] Patel DD, Dave RI (1976). Carcinoma of the anterior tongue in adolescence. Cancer.

[19] Harper JI, Copeman PW (1981). Carcinoma of the tongue in a boy with xeroderma pigmentosum. Clin Exp Dermatol.

[20] Yagi K, Ali AE, Abbas KE (1981). Carcinoma of the tongue in a patient with xeroderma pigmentosum. Int J Oral Surg.

[21] Kennedy AW, Hart WR (1982). Multiple squamous-cell carcinomas in Fanconi’s anemia. Cancer.

[22] Nuutinen J, Kärjä J, Sainio P (1982). Epithelial second malignant tumours in retinoblastoma survivors. A review and report of a case. Acta Ophthalmol.

[23] Newman AN, Rice DH, Ossoff RH (1983). Carcinoma of the tongue in persons younger than 30 years of age. Archi of otolaryngol.

[24] McGregor GI, Davis N, Robins RE (1983). Squamous cell carcinoma of the tongue and lower oral cavity in patients under 40 years of age. Am J Surg.

[25] Son YH, Kapp DS (1985). Oral cavity and oropharyngeal cancer in a younger population. Review of literature and experience at Yale. Cancer.

[26] Kaplan MJ, Sabio H, Wanebo HJ (1985). Squamous cell carcinoma in the immunosuppressed patient: Fanconi’s anemia. Laryngoscope.

[27] Sacks HG, Holly R, Blum B (1985). Case 58: maxillary alveolar mass in a 13-year-old boy. J Oral Maxillofac Surg.

[28] Usenius T, Karja J, Collan Y (1987). Squamous cell carcinoma of the tongue in children. Cancer.

[29] Earle AS, Park CH, Vlastou C (1988). Oral squamous cell carcinoma in children. Ann Plast Surg.

[30] Kärjä J, Syrjänen S, Usenius T (1988). Oral cancer in children under 15 years of age. A clinicopathological and virological study. Acta Otolaryngol Suppl.

[31] Amichetti M (1989). Squamous cell carcinoma of the oral tongue in patients less than fifteen years of age: report of a case and review of the literature. J Cranio-Maxillofacial Surg.

[32] Valerie JL, David JH (1990). Head and neck cancer in the young: a prognostic conundrum?. J Laryngology Otology.

[33] Murayama S, Manzo RP, Kirkpatrick DV, et al. Squamous cell carcinoma of the tongue associated with Fanconi’s anemia: MR characteristics. Pediatr Radiol. 1990;20(5):347. 10.1007/BF02013172 2349018

[34] Socié G, Henry-Amar M, Cosset JM (1991). Increased incidence of solid malignant tumors after bone marrow transplantation for severe aplastic anemia. Blood.

[35] Morehead JM, Parsons DS, McMahon DP (1993). Squamous cell carcinoma of the tongue occurring as a subsequent malignancy in a 12-year-old acute leukemia survivor. Int J Pediatr Otorhinolaryngol.

[36] Tsukuda M, Ooishi K, Mochimatsu I (1993). Head and neck carcinomas in patients under the age of forty years. Jpn J Cancer Res.

[37] Sarkaria JN, Harari PM. Oral tongue cancer in young adults less than 40 years of age: rationale for aggressive therapy. Head Neck. 1994;16(2):107–11. 10.1002/hed.2880160202 8021128

[38] Somers GR, Tabrizi SN, Tiedemann K (1995). Squamous cell carcinoma of the tongue in a child with Fanconi anemia: a case report and review of the literature. Pediatr Pathol Lab Med.

[39] Atula S, Grénman R, Laippala P (1996). Cancer of the tongue in patients younger than 40 years. A distinct entity?. Arch Otolaryngol Head Neck Surgery.

[40] de Carvalho MB, Sobrinho JDA, Rapoport A (1998). Head and neck squamous cell carcinoma in childhood. Med Pediatr Oncol.

[41] Jin YT, Myers J, Tsai ST (1999). Genetic alterations in oral squamous cell carcinoma of young adults. Oral Oncol.

[42] Effrey NM, Tina E, Dianna R (2000). Squamous cell carcinoma of the tongue in young adults: increasing incidence and factors that predict treatment outcomes. Otolaryngol Head Neck Surg.

[43] Torossian JM, Beziat JL, Philip T (2000). Squamous cell carcinoma of the tongue in a 13-year-old boy. J Oral Maxillofac Surg.

[44] Oliver RJ, Dearing J, Hindle I (2000). Oral cancer in young adults: report of three cases and review of the literature. Br Dent J.

[45] Pitman KT, Johnson JT, Wagner RL (2000). Cancer of the tongue in patients less than forty. Head Neck.

[46] Bill TJ, Reddy VR, Ries KL (2001). Adolescent gingival squamous cell carcinoma: report of a case and review of the literature. Oral Surg Oral Med Oral Pathol Oral Radiol Endod.

[47] Soni S, Radel E, Smith RV (2001). Stage 4 squamous cell carcinoma of the tongue in a child: complete response to chemoradiotherapy. J Pediatr Hematol Oncol.

[48] Wang Y, Irish J, MacMillan C (2001). High frequency of microsatellite instability in young patients with head-and-neck squamous-cell carcinoma: lack of involvement of the mismatch repair genes hMLH1 AND hMSH2. Int J Cancer.

[49] Abdelsayed RA, Sumner T, Allen CM (2002). Oral precancerous and malignant lesions associated with graft-versus-host disease: report of 2 cases. Oral Surg Oral Med Oral Pathol Oral Radiol Endod.

[50] Annertz K, Anderson H, Biörklund A (2002). Incidence and survival of squamous cell carcinoma of the tongue in Scandinavia, with special reference to young adults. Int J Cancer.

[51] Hyam DM, Conway RC, Sathiyaseelan Y (2003). Tongue cancer: do patients younger than 40 do worse?. Aust Dent J.

[52] Iamaroon A, Pattanaporn K, Pongsiriwet S (2004). Analysis of 587 cases of oral squamous cell carcinoma in northern Thailand with a focus on young people. Int J Oral Maxillofacial Surg.

[53] Sturgis EM, Moore BA, Glisson BS (2005). Neoadjuvant chemotherapy for squamous cell carcinoma of the oral tongue in young adults: a case series. Head Neck.

[54] Sasaki T, Moles DR, Imai Y (2005). Clinico-pathological features of squamous cell carcinoma of the oral cavity in patients < 40 years of age. J Oral Pathol Medicine.

[55] Salum FG, Martins GB, de Figueiredo MAZ (2006). Squamous cell carcinoma of the tongue after bone marrow transplantation in a patient with fanconi anemia. Braz Dent J.

[56] O’regan EM, Timon C, Sheils O (2005). Squamous cell carcinoma of the head and neck in young Irish adults. Br J Oral Maxillofacial Surg.

[57] Oluseyi FA, Wasiu LA, Akinola LL (2007). Malignant orofacial neoplasms in children and adolescents: a clinicopathologic review of cases in a Nigerian tertiary hospital. Int J Pediatr Otorhinolaryngol.

[58] Chung WC, Sepher NT, Karin T (2007). Squamous cell carcinomas in children and young adults: a new wave of a very rare tumor?. J Pediatr Surg.

[59] Reinhard H, Peters I, Gottschling S (2007). Squamous cell carcinoma of the tongue in a 13-year-old girl with Fanconi anemia. J Pediatr Hematol Oncol.

[60] Binahmed A, Charles M, Campisi P (2007). Primary squamous cell carcinoma of the maxillary alveolus in a 10-year-old girl. J Can Dent Assoc.

[61] S. A H S, A. G D, E. H V D M, et al. Oral squamous cell carcinoma in children; review of an unusual entity. Int J Pediatr Otorhinolaryngol. 2007;72(1):127–31. 10.1016/j.ijporl.2007.09.006 18029030

[62] Olajumoke AE, Wasiu LA, Olufemi GO, et al. Oral squamous cell carcinoma: a clinicopathologic review of 233 cases in Lagos, Nigeria. J Oral Maxillofac Surg. 2007;66(8):1595–9. 10.1016/j.joms.2007.12.025 18634945

[63] Masserot C, Peffault DLR, Rocha V (2008). Head and neck squamous cell carcinoma in 13 patients with Fanconi anemia after hematopoietic stem cell transplantation. Cancer.

[64] Randhawa T, Shameena P, Sudha S (2008). Squamous cell carcinoma of tongue in a 19-year-old female. Indian J Cancer..

[65] Mehanna P, Patel J, Bailey BMW (2008). Mandibular SCC in a 10 year old child – a clinical rarity. Br J Oral Maxillofacial Surg.

[66] Alsharif MJ, Jiang WA, He S (2009). Gingival squamous cell carcinoma in young patients: report of a case and review of the literature. Oral Surg Oral Med Oral Pathol Oral Radiol Endod.

[67] Sidell D, Nabili V, Lai C (2009). Pediatric squamous cell carcinoma: case report and literature review. Laryngoscope.

[68] Bachar G, Hod R, Goldstein DP (2010). Outcome of oral tongue squamous cell carcinoma in patients with and without known risk factors. Oral Oncol.

[69] Hutton A, McKaig S, Bardsley P (2010). Oral carcinoma cuniculatum in a young child. J Clin Pediatr Dent.

[70] Fadoo Z, Naz F, Husen Y (2010). Squamous cell carcinoma of tongue in an 11-year-old girl. J Pediatr Hematol Oncol.

[71] Nagy A, Barabas J, Vannai A (2010). Report of a rare case of tongue cancer in childhood. Orv Hetil.

[72] Morris LGT, Patel SG, Shah JP, et al. Squamous cell carcinoma of the oral tongue in the pediatric age group: a matched-pair analysis of Survival SCC of oral tongue in pediatric patients. Arch Otolaryngol Head Neck Surg. 2010;136(7):697–701. 10.1001/archoto.2010.94 PMC3005582 20644066

[73] Moubayed SP, Chami R, Woods O (2011). Neonatal squamous cell carcinoma of the lip: case report and review of the literature. J Clin Oncol.

[74] Ribeiro CM, Gueiros LA, Leon JE (2011). Oral squamous cell carcinoma in a 7-year-old Brazilian boy. Int J Oral Maxillofac Surg.

[75] Iraj H, Sayyedmohammadreza H, Sayyedmortaza K (2012). Childhood tongue squamous cell carcinoma. J Res Med Sci.

[76] Aaron S, Dana P, Sandeep S (2014). Pediatric mandibular reconstruction following resection of oral squamous cell carcinoma: a case report. Am J Otolaryngology-Head Neck Med Surg.

[77] Supriya VB, Suman MB (2015). Pediatric head and neck squamous cell carcinoma: report of 12 cases and illustrated review of literature. Int J Pediatr Otorhinolaryngol.

[78] Smile K, Ankit A (2019). Carcinoma of tongue in xeroderma pigmentosum a medical image. Imaging Med.

[79] Ambele MA, Pepper MS, van Heerden MB (2019). Molecular profile of tongue cancer in an 18-year-old female patient with no recognizable risk factor[J]. Laryngoscope Investig Otolaryngol.

[80] Best DL, Spresser W, Shivers P (2020). Squamous cell carcinoma of the tongue in young patients: a case series and literature review. J Oral Maxillofac Surg.

[81] Mendonça RMH, Cappellaro KMC, Gueiros LA (2021). Tongue carcinoma as a secondary malignancy in a 17-year-old leukemia survivor: a case report. Spec Care Dentist.

[82] Jh K, Cs K (2021). Squamous cell carcinoma of the tongue in 5-year-old girl with dyskeratosis congenita. Int J Oral Maxillofac Surg.

[83] Pereira TDSF, Castro LP, Menck CFM (2021). Xeroderma pigmentosum variant: squamous cell carcinoma of the lower lip harboring exon 11 mutation of POLH. Oral surgery, oral medicine, oral pathology and oral radiology.

[84] Medeus AJR, Desravines AJ, Cotard V (2022). Verrucous carcinoma of tongue in Xeroderma Pigmentosum: a case report and literature review. Cureus.

[85] Sahin EA, Taskiran EZ, Kiper P (2022). Recurrent squamous cell carcinoma and a novel mutation in a patient with xeroderma pigmentosum: a case report. J Med Case Rep.

[86] Yabe TE, King K, Russell S (2022). MYH9 mutation and squamous cell cancer of the tongue in a young adult: a novel case report. Diagn Pathol.

[87] Kikuta S, Teratani Y, Matsuo K (2023). Gingival squamous cell carcinoma predicted to originate from the gingival sulcular epithelium in a young female: a report of a rare case. Cureus.

[88] Okuyama K, Yanamoto S, Michi Y (2021). Multicenter retrospective analysis of clinicopathological features and prognosis of oral tongue squamous cell carcinoma in adolescent and young adult patients. Med (Baltim).

[89] Johnson DE, Burtness B, Leemans CR (2020). Head and neck squamous cell carcinoma. Nat Rev Dis Primers.

[90] Ferreira DA, McMillan N, Idris A (2022). Genetic deletion of HPV E7 oncogene effectively regresses HPV driven oral squamous carcinoma tumour growth. Biomed Pharmacother.

[91] Lewis JJ, Smith MH, Wang X (2022). Human papillomavirus-associated oral cavity squamous cell carcinoma: an entity with distinct morphologic and clinical features. Head Neck Pathol.

[92] Caley A, Evans M, Powell N (2015). Multicentric human papillomavirus-associated head and neck squamous cell carcinoma. Head Neck.

[93] Ang KK, Harris J, Wheeler R (2010). Human papillomavirus and survival of patients with oropharyngeal cancer. N Engl J Med.

[94] Jiang S, Dong Y (2017). Human papillomavirus and oral squamous cell carcinoma: a review of HPV-positive oral squamous cell carcinoma and possible strategies for future. Curr Probl Cancer.

[95] Kouketsu A, Sato I, Abe S (2016). Detection of human papillomavirus infection in oral squamous cell carcinoma: a cohort study of Japanese patients. J Oral Pathol Med.

[96] Oh KY, Cho SD, Yoon HJ (2019). High prevalence of BRAF V600E mutations in Korean patients with ameloblastoma: clinicopathological significance and correlation with epithelial-mesenchymal transition. J Oral Pathol Med.

[97] Dombrowski ND, Wolter NE, Irace AL (2019). Squamous cell carcinoma of the head and neck in children. Int J Pediatr Otorhinolaryngol.

[98] Amin MB, Greene FL, Edge SB (2017). The eighth edition AJCC cancer staging manual: continuing to build a bridge from a population-based to a more personalized approach to cancer staging. CA Cancer J Clin.

